# Novel analytic tools for the study of porcine reproductive and respiratory syndrome virus (PRRSv) in endemic settings: lessons learned in the U.S.

**DOI:** 10.1186/s40813-016-0019-0

**Published:** 2016-01-21

**Authors:** Julio Alvarez, Pablo Valdes-Donoso, Steven Tousignant, Mohammad Alkhamis, Robert Morrison, Andres Perez

**Affiliations:** 1 Department of Veterinary Population Medicine, College of Veterinary Medicine, University of Minnesota, St. Paul, MN USA; 2 Department of Agriculture and Resource Economics, University of California Davis, Davis, CA USA; 3 Environmental and Life Sciences Research Center, Kuwait Institute for Scientific Research, Kuwait City, Kuwait

**Keywords:** PRRS, epidemiology, control, regional projects, production, phylogenetics

## Abstract

Since its emergence in the late 1980’s, the porcine reproductive and respiratory syndrome virus (PRRSv) has posed a significant challenge to the pig industry worldwide. Since then, a number of epidemiological tools have been created to support control and eventual elimination of the disease at the farm and regional levels. Still, many aspects of the disease dynamics are yet-to-be elucidated, such as what are the economically optimal control strategies at the farm and regional level, what is the role that the voluntary regional control programs may play, how to optimize the use of molecular tools for surveillance and monitoring in infected settings, what is the full impact of the disease in a farm, or what is the relative contribution of alternative transmission routes on the occurrence of PRRSv outbreaks. Here, we summarize a number of projects demonstrating the use of novel analytical tools in the assessment of PRRSv epidemiology in the United States. Results presented demonstrate how quantitative analysis of routinely collected data may help in understanding regional epidemiology of PRRSv and to quantify its full impact, and how the integration of phylodynamic methods as a standard tool for molecular surveillance of PRRSv might help to inform control and prevention strategies in high-risk epidemiological situations. Ultimately, these tools will help to support PRRSv control at farm and regional levels in endemically infected settings.

## Background

Since its emergence in the late 1980s, the porcine reproductive and respiratory syndrome virus (PRRSv) has posed a significant challenge to the pig industry worldwide. Even though PRRS is a relatively new disease affecting swine, its rapid worldwide spread, the lack of efficient control tools in the first years after its discovery, and its complex epidemiology and pathogenesis resulted in a number of published scientific papers comparable to that published during a longer period for other swine diseases. However, despite substantial efforts and resources invested by researchers worldwide, there are still many important gaps in our understanding of critical aspects of its epidemiology [1]. Those unknowns include, for instance, an updated knowledge of regional PRRSv dynamics in endemically infected areas, full quantification of its impact on production (particularly in subclinically affected herds), and development of improved tools for assessing the relation between PRRSv strains with a close genetic relatedness and linked epidemiologically at a local level. There are, nevertheless, new opportunities to address those problems thanks to the increasing awareness of the value of sharing information to improve disease management at a supra-farm level [2], the extended use of production management software that allows recording and storage of large datasets over long periods of time, development of standardized and optimized cost-effective diagnostic systems for surveillance that may be linked with production data [3, 4], and the increasing availability of molecular tools [5]. This changing reality is expected to lead to the generation of information that may be then combined with novel analytical tools to gain new insights on the epidemiology of PRRS. The objective of the paper here is to summarize the work that has been done to provide answers to some of those pending questions on PRRS epidemiology with a focus on studies performed in the U.S., where the predominant PRRSv type is type 2 [6]. We initially focus on PRRSv control at the regional level, then we review the use of routinely collected production data to evaluate the impact of PRRS at the system or farm level, and finally we introduce the potential application of molecular tools to the near real time surveillance of virus spread. The review here will help to understand how quantitative analysis of routinely collected data may help to design effective strategies to support PRRS control at the regional and local levels, and ultimately, improve health status in swine farms and systems.

## Review

### Quantitative evaluation of control strategies for PRRSv at the regional level

Implementation of mandatory control programs has resulted in the eradication of numerous swine diseases in the U.S., such as classical swine fever [7, 8] and Aujeszky’s disease [9, 10]. Despite the early adoption of a voluntary scheme for PRRS control [1], the disease has remained endemic [11–13], and represents one of the major production diseases causing more than $600 million annual losses [14]. Among the currently available strategies to prevent or control PRRSv infection and impact, intentional exposure of the population in sow farms to the virus (either using modified live vaccine or field strains) and elimination of positive sows using test-and-removal strategies (with or without complementary herd closure and rollover) have been evaluated as effective measures for on-site PRRSv control or eradication [1, 12, 15–18]. Other actions, including use of air filtration systems, enhanced biosecurity measures, and quarantine of new gilts may help to decrease the risk of new virus introductions [19–22]. In nursery and grow-to-finish sites, the use of strategies based on all-in-all-out management, testing of newly introduced animals, therapeutic vaccination, and elimination of sick animals for PRRSv control has been also evaluated [21–24]. The scientific literature thus demonstrates that significant achievements towards the development of strategies for the control of PRRS at the herd level have been achieved in the last decades, but the extent to which those advancements have been translated into progress at a large geographical scale is unclear. In this context, analysis of data at the supra-herd level may help to understand disease trends and to evaluate the progress of control and eradication strategies currently in place.

**Fig. 1:**
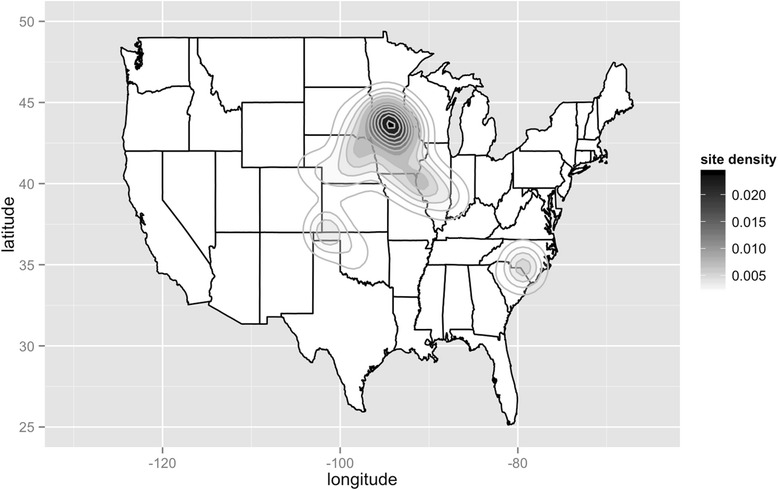
Plot of geographical density of swine farms currently enrolled in the Swine Health Monitoring Project (SHMP) in the United States

Analysis of data collected as part of the Swine Health Monitoring Project (SHMP), a voluntary project monitoring the incidence of PRRS in a cohort of sow farms (still ongoing and currently including 742 farms with approximately 2 million sows, i.e., approximately one third of the U.S. sow population) (Fig. 1) that began in 2011, has demonstrated that the incidence of PRRS has shown a repeatable pattern between 2009 and 2013 [25]. During this period, PRRS annual incidence consistently increased during the months of September through November and decreased during the months of February through April, with epidemic levels of disease [established using an Exponentially Weighted Moving Average (EWMA) chart method] being reached in the middle of October [25]. Among the 371 sow herds in the database at that time, it was shown that 29 – 38 % of the herds reported a new PRRS infection each year [25]. Additionally, this study identified significant spatial clustering in swine dense regions of Minnesota and Iowa, further reinforcing the need for a comprehensive approach to make an impact in the disease at the regional level [25]. Analysis of the same cohort of sow farms revealed a significant decrease in PRRS incidence In 2013-2014, when the PED epidemic spread within the U.S. swine population, in comparison with previous years [26]. It was suggested that improved biosecurity measures aimed at preventing PED transmission may have also reduced PRRS incidence, in addition to increased PRRS vaccine use and awareness of the annual epidemics [26]. That study also showed the extent of the spatial clustering of disease was similar to the previous years, suggesting certain factors within those regions may be contributing to these observations [26].

The difficulty controlling PRRS across large areas of the U.S., combined with the absence of an official regulatory framework that provides guidelines to control PRRS, has triggered the organization of numerous producer-led regional strategies. Regional control and elimination programs for PRRSv have demonstrated to be effective in regions sporadically affected by the disease outside North America [27–29], but their impact in endemic settings had not been quantified. In the U.S., the N212 regional control project (N212-RCP), which was started by a group of producers in Stevens County (Minnesota) in 2004, was among the first voluntary RCPs launched to control PRRSv infection in the country. This approach has been followed in other North American regions since then, so that currently there are more than 30 RCPs throughout the U.S. and Canada [30]. In the context of a highly heterogeneous and complex industry, RCPs have promoted sharing PRRS, and lately Porcine Epidemic Diarrhea (PED), status among producers, with the expectation that such information may help to control disease within a geographical area [9, 30, 31]. Within RCPs, the various strategies to control or eliminate PRRSv will depend on the type of farm (i.e. farms with or without sows) and have been traditionally focused on farms with sows, aiming to wean PRRSv-negative piglets [1, 12]. Even though there is a general perception that RCPs have been beneficial on PRRS control, the extent to which this statement is true has not been formally assessed. For this reason, a study to quantify if RCPs have contributed to PRRS control was conducted using data from the RCP-N212 from June 2012 to July 2014 as a working model. This study included the development of a methodological framework to evaluate the progress of an RCP, anticipating the establishment of a benchmark, which may assist comparisons among RCPs (Valdes-Donoso et al., submitted). Number of farms enrolled in the RCP-N212 and geographical coverage increased over time during the study period, and an increasing proportion of farms shared PRRS status throughout the 24 months, though farms without sows (approximately 77 % of all farms) were less willing to report their status; interestingly, a significantly higher variability in the communication pattern between counties than within counties was observed, suggesting that farms were more influenced by their neighboring sites. PRRS incidence decreased significantly (P < 0.001) over the study period regardless of the site type, but despite this decreasing trend of PRRS incidence, repeated clusters of increased risk of disease were demonstrated within 3 weeks and 3 km of other incident cases, suggesting an important role of local factors in disease spread (Valdes-Donoso et al., submitted).

A limitation common to these studies, performed at different geographical scales [with a database with national (SHMP) and regional (RCP-N212) coverages] is the lack of information on a proportion of the farms present in the area. Still, a remarkably significant spatial and spatio-temporal pattern was identified at both levels, and the value of the information extracted from these studies for the stakeholders is evidenced by the increasing number of enrolled participants at both levels, what in turn has and will continue to increase the power of the analysis.

### Use of production records to quantify the impact of PRRSv in production

Quantifying the impact of infectious livestock diseases is challenging given the many different ways in which its effect may be measured. This includes direct mortality, increased production/days to achieve a given amount, economic revenue per head/kg of product, and use of treatments or diagnostics. Even though there is an increasing body of evidence on successful strategies for controlling and eventually eradicating PRRSv at the herd level [12, 16, 32], a thorough evaluation of the impact of the disease at all levels of the production chain is required to identify cost-effective approaches to control the disease [33]. Such evaluation is, however, particularly challenging due to the broad variation in the severity and duration of the clinical symptoms that can occur in infected farms, potentially associated with different strains and management-dependent factors [34, 35]. In addition, the use of different methodologies for assessment of the impact of the disease (comparison of production records from the same farm before and after an outbreak versus comparison of records from affected and unaffected farms with similar characteristics) can be an additional source of variability of the outcomes of the studies.

#### Impact of PRRSv infection in breeding farms

In the breeding herd, impact of PRRSv infection is mainly due to an increased rate of reproductive failures (abortions, stillbirths, weak piglets, delayed return to estrus and low conception rates in following inseminations of infected sows), although adult animals can be also affected (mainly in acute outbreaks) [36]. Costs associated with decreased production (decrease in the number of weaned pigs) and increase animal health needs may be highly variable depending on several factors, and have been reported to range during acute outbreaks in the US between $100 and $510 per sow, with a mean value of $255 (reviewed in [37]). The magnitude of the decrease in the farrowing rate and the number of pigs weaned per sow farrowed in clinically affected farms can be also variable, with percentage changes (compared with records from the same farm before the outbreak or PRRS-unaffected farms belonging to the same system) in a cohort of 9 sow farms ranging between 2 and 39 % (farrowing rate, mean value = 13.8 %) and 6-33 % (pigs weaned/sow farrowed, mean percentage change = 16.4 %) [13]. A similar study comparing the productivity (piglets born alive, pre and post-weaning mortality) of nine sow herds in the 26 weeks before and 18 weeks after experiencing a PRRSv outbreak in the Netherlands described a mean percentage decrease of 8 % in the litter size, and a mean percentage increase in pre and post-mortality of 36 and 167 %, respectively (absolute increase of 5.1 and 2 percentage points, respectively) [38]. Again, a large variability in effect of the outbreaks at the herd-level was described, with two herds even reported an unexplained increase in their farrowing index during the outbreak [38]. Interestingly, these two latter studies provided similar estimates of the mean cost of an 18-weeks PRRSv outbreak per affected sow (combining the cost associated with decreased litter sizes and increased cost of weaned pigs) of $121 and €126 ($137) [13, 38].

However, measurement of impact beyond the acute (clinical) phase of the disease is often more challenging due to the lack of standardized criteria to define when the post-outbreak period may begin and end, and the need for longitudinal data with a meaningful timeframe. When time to return to historical values of production variables is used to define outbreak duration lengths between 2 and 28 months have been described [13, 35, 38, 39]. A more formal approach to define the time-to-baseline production (TTBP) after a PRRSv outbreak in sow farms was recently proposed by Linhares and others, using weekly number of weaned piglets as the indicator and the EWMA control chart method to adjust by the previous performance of the farms (previous 21 weeks) [40]. Additionally, laboratory test results can help to define more precisely the ending point of an outbreak, though they may not correlate with certain productive indicators as the number of weaned pigs [40]. Consistent lack of positive PCR results in batches of pigs weaned consecutively and of seroconversion in external gilts/sows negative prior to their introduction in the herd can be used to establish the shedding and exposure status of a breeding herd [3]. This information has been used to evaluate the impact of PRRSv infection in sow herds depending on their previous history (occurrence of one or more outbreaks in the 12 months previous to the present outbreak based on laboratory results), suggesting that infected farms that had remained negative in the 12 months before an outbreak outperformed farms with exposure to the virus in that timeframe in terms of pigs farrowed alive and pre-weaning mortality. In contrast, for those farms exposed in the previous 12 months, farms that were considered to be PRRSv-free in the moment of the new outbreak had poorer performance for those two indicators that those that were not PRRSv-negative immediately before the new PRRSv outbreak [41]. Alternatively, the time elapsed before weaning negative pigs as defined above (time to stability, TTS) and the TTBP have been used as the response variable in models aiming at the evaluation of the effect of different control strategies (exposure to vaccine or live-resident viruses) adjusting by other potential confounders [40]. This study also revealed a significant effect of the occurrence of outbreaks of PRRSv in the previous three years, leading to a significantly shorter duration of the following one, and that use of live-resident virus for whole-herd exposure was associated with shorter TTS and TTBP than commercial vaccination. Preliminary evidence in a cohort of 60 sow farms suggest that shorter TTS may be consistently achieved in certain farms in subsequent outbreaks, possibly pointing at farm-level associated factors, such as biosecurity measures, or whole herd exposure programs.

#### Impact of PRRSv infection in growing farms

Fewer number of studies have focused on the impact of PRRSv infection in the nursery/growing/finishing stages, where disentangling its effect from that due to other common pathogens after weaning, such as Influenza A viruses, *Mycoplasma hyopneumoniae*, *Streptococcus suis*, *Actinobacillus pleuropneumoniae, Salmonella cholerasuis,* is a challenge [36, 42–44]. Although some estimates of the economic impact of PRRS in growing pigs have been published (ranging from $0.7 to $18.2 per pig [37]) values vary with the epidemiological conditions of the herd, making it difficult to extrapolate results to other conditions. In addition, they often rely on the use of certain assumptions of baseline performance that may not be applicable to all production systems. Comparison of production records from PRRSv-positive and PRRSv-negative batches of growing pigs from different farms and production systems suggested that infection could lead to a mean increase of mortality and decrease in average daily gain (ADG) and feed efficiency of 11, 25 and 12 % in nursery pigs and 6, 12 and 8 % in growing pigs respectively [13]. Even though a limited number of farms was included in the study (n = 8), a large between-farm variability was identified, highlighting the potential effect of farm-level factors. The use of clinical signs as a prerequisite for defining a batch as positive in this study could have ruled out endemically infected herds with a more subtle clinical presentation, thus potentially biasing the results [13]. A similar analysis performed on a dataset that included growing pigs from six production systems in which PRRSv-status was defined based on laboratory data (serology at weaning and marketing) provided a more limited but still significant estimate of the effect of infection. In this study a significantly increased mortality was observed in groups of pigs positive both at weaning and marketing (9.3 %) compared to positive just at marketing (7.4 %) or negative at both samplings (6 %), and a significantly lower ADG in animals positives at either sampling point with respect to negative batches was found, while feed efficiency was not affected [41]. Another recent study using close-out data from 177 batches of growing pigs originated in 9 sow farms from one production system in the Midwest region of the U.S. detected a statistically important effect of PRRS status at weaning in post-weaning mortality (with positive batches having a median percentage increase over the system-baseline mortality of 34 %) [45].

Herd and production system are often included as random effects or hierarchies in the analytical models used to measure disease impact on production. However, the only fixed effects (in addition to PRRS status) for which, usually, there are available data to account for their effect are usually time of placement and slaughtering of growing pigs (month/year/season) and batch-specific characteristics (days on feed, size) [41, 45]. Absence of data on variables likely to influence the outcome, such as history and management of PRRSv in the breeding herds from which growing pigs originated and presence of co-infection with other pathogens, limits the interpretation of results. Even though management factors play a major role in post-weaning mortality [46] and can be at least partially accounted for in mixed models, co-infection with other pathogens is likely another critical source of the reported variability on the effect of PRRSv in growing pigs. Unfortunately, the lack of confirmation of the presence (and eventually, the prevalence) of other diseases in the growing site impairs the ability to assess the relation (synergies, antagonism) between them. Because sow farms are usually subjected to more strict surveillance protocols than growing sites, detection of other pathogens at weaning (in the frame of ongoing surveillance programs) could be used as a cost-effective proxy of their presence in the growing phase, but a sufficiently large sample size would be required in order to assure the statistical power needed to assess potential interactions between them [45].

### Use of evolutionary epidemiology for surveillance of PRRS in endemic settings

PRRSv is a single stranded enveloped virus with an RNA genome that consists of nine open reading frames (ORF) which code seven structural proteins and 14 non-structural proteins [47]. ORF5 encodes a major envelope surface glycoprotein (GP5), which plays an important role in viral infection and antigenicity [48, 49]. Therefore, ORF5 has been widely used in molecular epidemiology studies of PRRSv. In the past decade, a large number of PRRSv genomic sequences became available due to the increasing availability of affordable molecular tests. This extensive availability of data has resulted in new challenges in the interpretation of the results due to the frequent occurrence of mutation events and the high recombination rates of the virus, and highlighted the need for objective criteria to establish when two strains should be considered epidemiologically related in an endemic setting [50, 51].

Many studies on the evolutionary epidemiology of PRRSv have been attempted with the ultimate goals of assessing the evolutionary patterns of the virus and guiding the decision making process related to the control and prevention resources. Some studies focused on establishing association between the evolutionary features of PRRSv and epidemiological characteristics of outbreaks in different geographical levels [52–57]; others have focused on discriminating between novel and preexisting strains to model their spread and maintenance within affected populations [19, 58–60]. However, most of those observational studies used classical phylogenetic methods to either genotype newly emerging PRRSv strains on the basis of restriction fragment length polymorphism (RFLP) patterns [19, 59, 61], or assess correlations between the similarities of nucleotide sequences and other epidemic features [19, 52, 57, 62]. Unfortunately, those methods typically ignore various sources of uncertainty, including that associated with estimates of the phylogenetic relationships, such as branch lengths and substitution rates. Furthermore, they assess the temporal and spatial dynamics of the virus isolates in separate methodological settings, and attempt to draw conclusions from the outputs of both epidemiological and evolutionary analytical methods [63]. Therefore, previous methodological approaches ignore that evolutionary and epidemiological dynamics of rapidly evolving pathogens like PRRSv occur on approximately the same time-scale, and thus, they must be studied in a unified methodological setting in order to be properly understood and to prevent biased conclusions, subsequently improving the related decision making processes [64]. Phylodynamics is an emerging field that aims to characterize the joint evolutionary and epidemic behavior of rapidly evolving infectious diseases using tools borrowed from the field of phylogenetics in a Bayesian statistical framework [65]. This approach treats parameters of the phylodynamic model as random variables, such that each parameter is described by a specified prior probability distribution (and a corresponding inferred posterior probability distribution). Accordingly, the Bayesian approach provides a natural way to estimate (and accommodate) uncertainty in the phylodynamic model parameters, including the virus phylogeny, divergence times, and history of geographic spread [66]. Bayesian models have been demonstrated, for example, that the difference in the number of nucleotides between PRRSv sequences is an inaccurate measure of true phylogenetic distance [58].

Bayesian phylodynamic models are becoming a well-established method for the study of the evolution of many infectious animal and human viral pathogens like avian influenza [67] or Ebola [68]. However, few studies have attempted to model the evolutionary dynamics of PRRSv [54, 56, 58, 69]. Still, those studies have revealed the potential for answering some major questions still unresolved about the evolutionary epidemiology of PRRSv of phylodynamic methods belonging to three different domains:

- The first are *Bayesian coalescence models*, which have shown to provide robust inferences about the demographic histories and population growth patterns of viral lineages and sub-lineages [53]. The inclusion of information on nucleotide substitution schemes obtained from the data, allowing for different model assumptions to assess the degree of genetic relatedness under time-scaled phylogenies, has provided a robust strategy to distinguish between potentially related PRRSv strains detected in air samples and swine farms in areas of high and low density of swine farms [58]. This approach can help to shed further light on several evolutionary and epidemiological characters of endemic PRRSv and provide realistic basis for PRRSv genotyping.
- The second are *phylogeographic models*, which are extensions for the coalescence models, and have the ability to identify the ancestral geographical origins and spread epicenters of PRRSv on global and local levels [53, 56]. Those methods have demonstrated great potential for modeling temporal and the spatial dynamics of highly infectious viruses like PRRSv, including its dispersal patterns in endemic settings [70]. Inferences drawn from these models can be used to revise local and national hog transportation networks between and within production regions by overlapping viral dispersal routes with the transportation edges, which can lead to the identification of high-risk routes [70]. This could subsequently inform prevention and control measures to contain the virus at the source (e.g., high-risk geographical regions or swine herds), what can limit the spread of the virus both to naïve swine populations in infected settings and to uninfected geographical regions or areas. Furthermore, the ability to infer routes of viral transmission has clear implications for informing the selection of appropriate strains for vaccine production to control more effectively new virulent strains of PRRSv in future epidemics [70].
- The third are *​stochastic and deterministic coalescent susceptible-infected-removed (SIR) models* [71]; unlike common disease spread models, which depend on parameterization of observational count data as well as hypothetical scenario construction and simulation, those models have the ability to estimate important epidemiological characteristics from sequence data related to epidemic spread and progression such as the basic reproductive number (R_0_) and other related transmission rate parameters [72]. SIR phylodynamic models have demonstrated the ability to produce reliable estimates for the epidemic parameters of the Ebola virus, including infectious period duration and date of the first case, during the early stages of the its spread during the 2014-2015 outbreak in Sierra Leone [68]. Such inferences are essential in controlling epidemic progression of newly emerging PRRSv strains at early stages of their spread within geographical regions in the US, especially where sequences are currently more attainable than before during the initial phases of new epidemics.

Overall, these methodologies could provide additional insights to assess the genetic relatedness between strains and help to distinguish new from resident strains, and identify and evaluate quantitatively the likelihood of implication of different potential sources of infection at a farm level on a case-to-case basis, what could help to inform and optimize control and prevention strategies.

## Conclusions

Despite vast efforts that have been invested in fighting PRRSv infection, the disease is still present at a variable prevalence in many swine-producing regions of the world. Changes introduced in the swine industry in the U.S. and other countries during the second half of the 20^th^ century (increase in herd sizes, introduction of multi-site production systems, use of artificial inseminations) possibly contributed to its early dissemination and led to its endemicity in many swine densely populated areas [6], where eradication is particularly challenging. In fact, some of the main characteristics of today’s intensive swine production systems (large populations concentrated in small regions, high connectivity between sites, increasing level of genetic homogeneity among pig populations) may be particularly suited for the emergence of host-adapted RNA viruses (like PRRSv) in pigs [73, 74]. Recent estimates quantifying the impact of PRRS in the swine industry in the U.S. suggest that even though some progresses have been achieved, at a large scale the strategies implemented for its control have had little to no impact, and PRRS remains as a major problem for the U.S. industry [41]. All these factors suggest that the use of the farm as the epidemiological unit for the implementation of control strategies may lead to suboptimal results in the long term, particularly in highly populated regions, and that the coordination of efforts at a supra-farm level may be critical to success in the fight against the disease. In fact, some of the studies summarized here suggest that some of these strategies can be successful [12] (Valdes et al., submitted) and in at least a subset of the US swine industry a significant decrease in the incidence is achievable [26]. Certain characteristics in today’s swine industry can also help to achieve the control and eventual eradication of the virus. Awareness of the importance of collaborative efforts has increased significantly in the last years, and the experience gained fighting PRRSv and, more recently, PEDv in the U.S. has demonstrated the usefulness of sharing information for the improvement of the health of the national herd [2]. Studies reviewed here demonstrate how quantitative analysis of routinely collected data may help in understanding regional epidemiology of PRRSv and to quantify its full impact, which are pre-requisite the design, improvement and optimization of PRRSv control and eradication programs at the regional level. In addition, the integration of phylodynamic methods as a standard tool for molecular surveillance of PRRSv might support the development of effective (and efficient) policy decisions for the control and prevention of this virus in identified high-risk epidemiological settings. However, continued efforts will be needed to build on current achievements, in order to expand and update databases containing information on swine population and practices, and PRRSv incidence and genomic data, and to build efficient bioinformatics and computational infrastructures, basic requirements for the field of applied phylodynamics [75, 76].
